# Circulating histones as clinical biomarkers in critically ill conditions

**DOI:** 10.1002/1873-3468.70093

**Published:** 2025-06-17

**Authors:** José Luis García‐Gimenez, Juan Carlos Ruiz‐Rodríguez, Ricard Ferrer, Raquel Durá, Antonio Artigas, Iván Bajaña, David Bolado López de Andujar, Irene Cánovas‐Cervera, Adrián Ceccato, Luis Chiscano‐Camón, Elena Climent‐Martinez, Georgia García Fernández, Gemma Goma, Verónica Monforte, Beatriz Quevedo‐Sánchez, Adolf Ruiz‐Sanmartín, Antonio Sierra‐Rivera, Nieves Carbonell Monleón

**Affiliations:** ^1^ Department of Physiology, Faculty of Medicine and Dentistry University of Valencia Spain; ^2^ INCLIVA Biomedical Research Institute Valencia Spain; ^3^ Center for Biomedical Network Research on Rare Diseases (CIBERER) Carlos III Health Institute Valencia Spain; ^4^ Intensive Care Department Vall d'Hebron University Hospital, Vall d'Hebron Barcelona Hospital Campus Barcelona Spain; ^5^ Shock, Organ Dysfunction and Resuscitation Research Group. Vall d'Hebron Research Institute (VHIR), Vall d'Hebron Barcelona Hospital Campus Barcelona Spain; ^6^ Departament de Medicina Universitat Autonoma de Barcelona Spain; ^7^ Unidad de Cuidados Intensivos (UCI) de Anestesiología, Hospital General Universitario de Valencia Spain; ^8^ Institut d'Investigació i Innovació Parc Tauli (I3PT, CERCA) Barcelona Spain; ^9^ Critical Care Center, Sabadell Hospital Sabadell Spain; ^10^ Center for Biomedical Network Research on Respiratory Diseases (CIBERES) Barcelona Spain; ^11^ Medical Intensive Care Unit, Hospital Clínico Universitario de Valencia Spain

**Keywords:** acute kidney injury, cardiac injury, COVID‐19, critically‐ill patient, extracellular histones, lung injury, mass spectrometry, pancreatitis, renal replacement therapy, sepsis, septic shock

## Abstract

Extracellular histones, primarily nuclear proteins involved in chromatin organization, have emerged as key mediators in pathological processes in critically ill patients. When released into circulation due to cell death mechanisms such as NETosis, histones act as damage‐associated molecular patterns (DAMPs), contributing to excessive inflammation, endothelial dysfunction, immune response dysregulation, coagulation activation, cell death, and multi‐organ damage. Increasing evidence supports their role in the pathophysiology of sepsis, acute lung injury, cardiac injury, pancreatitis, and other life‐threatening conditions. Given their strong association with disease severity and prognosis, circulating histones have gained attention as potential clinical biomarkers for early diagnosis, prognosis, and therapeutic monitoring in critically ill patients. This review discusses the biological roles of extracellular histones, their potential as biomarkers, different approaches to measure them, and emerging therapeutic strategies aimed at neutralizing or removing circulating histones to improve patient outcomes in severe medical conditions.

**Impact statement**

This review highlights extracellular histones as key mediators and biomarkers in sepsis, proposing their use in diagnosis, prognosis, and treatment monitoring. Integrating quantitative proteomics for the detection of circulating histones may enhance patient stratification and guide therapeutic strategies, advancing personalized medicine in critical care.

## Graphical abstract

Circulating histones are emerging as promising biomarkers in critical illness due to their diagnostic, prognostic, and therapeutic potential. Detection methods such as ELISA and mass spectrometry provide reliable approaches for quantifying histone levels in plasma samples. Given their role in driving inflammation, cell death, and organ failure, therapeutic strategies targeting histone neutralization may help improve outcomes in critically ill patients. Created in BioRender. García‐Gimenez, J. (2025) https://BioRender.com/8vip33g

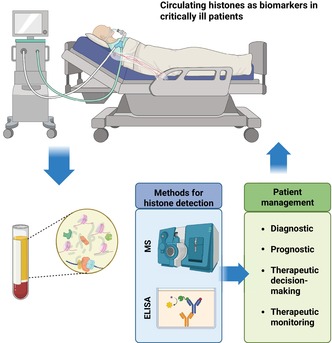

## Abbreviations

**AKI**, acute kidney injury

**AUC**, area under the curve

**cTn**, cardiac troponin

**CRP**, C‐reactive protein

**CRRT**, continuous renal replacement therapy

**DAMPs**, damage‐associated molecular patterns

**DIC**, disseminated intravascular coagulation

**ELISA**, Enzyme‐Linked Immunosorbent Assay

**ETosis**, extracellular trap cell death

**ICU**, intensive care unit

**IVD**, *in vitro* diagnostic

**LC–MS/MS**, Liquid Chromatography–Tandem Mass Spectrometry

**MS**, mass spectrometry

**NETs**, neutrophil extracellular traps

**NETosis**, neutrophil extracellular trap cell death

**PAMPs**, pathogen‐associated molecular patterns

**PCT**, procalcitonin

**PTMs**, posttranslational modifications

**ROS**, reactive oxygen species

**RUO**, research use only

**SIRS**, systemic inflammatory response syndrome

**Sn/Sp**, sensitivity/specificity

**SOFA**, sequential organ failure assessment

**TLR**, toll‐like receptor

The nucleosome serves as the fundamental structural unit of chromatin in all eukaryotic cells, playing a crucial role in genome organization and regulation. It consists of an octamer of highly conserved histone proteins, including two copies each of H2A, H2B, H3, and H4, around which approximately 145–147 base pairs of DNA wrap 1.65 times. These nucleosomes are interconnected by “linker DNA” segments ranging from 20 to 80 base pairs in length, with histone H1 stabilizing these linkages and contributing to chromatin compaction [1]. Beyond the primary function of histones and nucleosomes in DNA packaging, chromatin structure, and gene expression, histones have been recognized for their antimicrobial properties and immune‐modulating capabilities. However, under pathological conditions, the release of extracellular histones into the circulation can have severe detrimental effects, including exacerbation of inflammation, activation of coagulation pathways, tissue damage, and multi‐organ injury. Given their dual role in host defense and disease pathology, extracellular histones have emerged as key mediators of immune dysregulation, making them a significant research focus in inflammatory diseases such as sepsis and acute organ dysfunction [2].

**Fig. 1:**
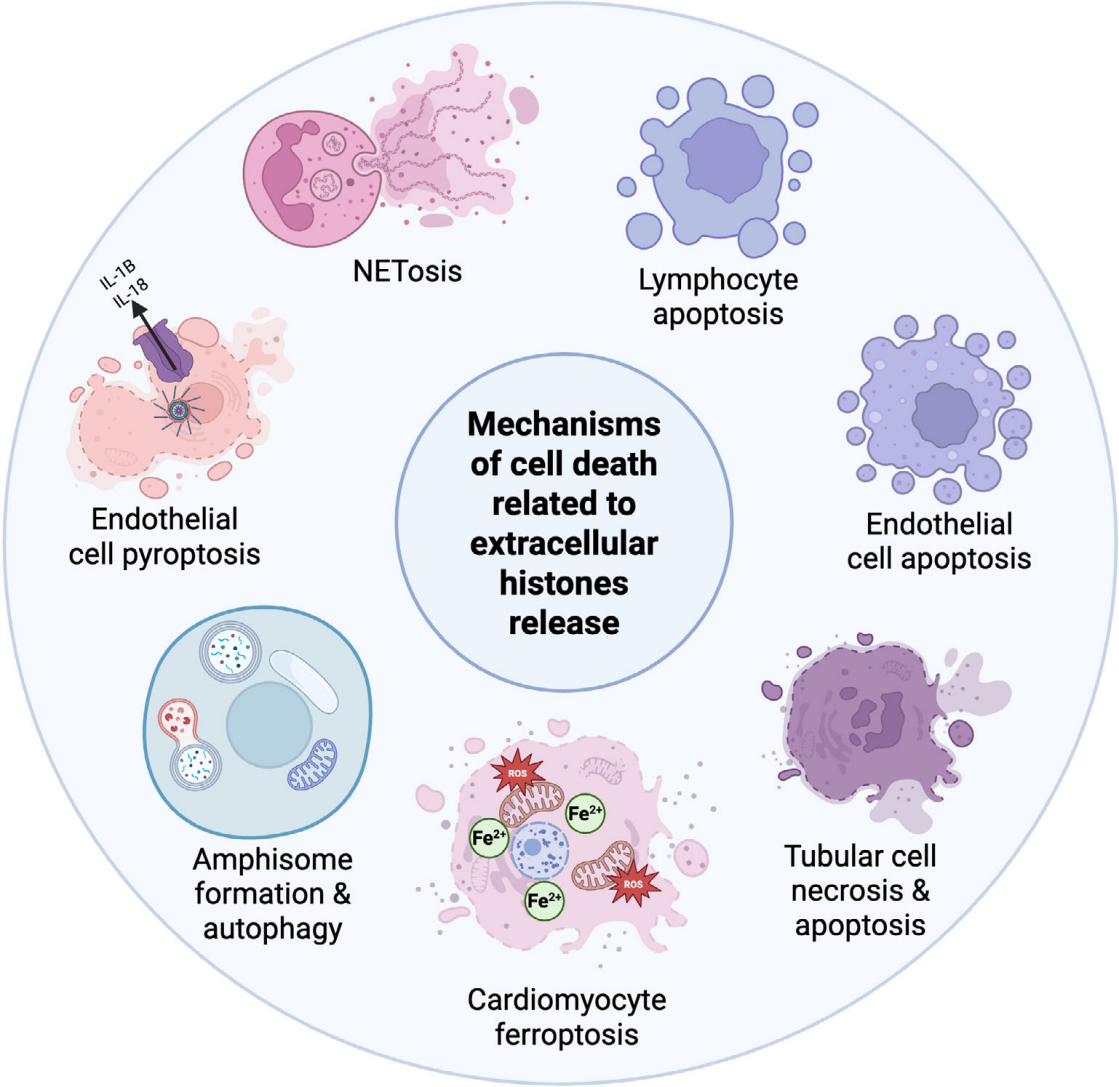
Mechanisms mediating the release of extracellular histones. Nuclear histones can be released from cells upon several cell death mechanisms, such as necrosis and necroptosis, apoptosis, pyroptosis, extracellular trap cell death (ETosis) of leukocytes, particularly neutrophil extracellular traps (NETosis), ferroptosis, and direct cell damage by pathogens or direct release through the formation of amphisomes. Notably, once released, histones can amplify the cytotoxic response by means of a positive feedback loop, further exacerbating tissue damage. Created in BioRender. García‐Gimenez, J. (2025) https://BioRender.com/mkat4dm

Nuclear histones can be released from cells upon several cell death mechanisms (Fig. 1), such as necrosis [3], apoptosis [4, 5], pyroptosis, extracellular trap cell death (ETosis) of leukocytes [6], particularly neutrophil extracellular traps (NETosis) [7], ferroptosis [8, 9], oncosis [10], and direct cell damage by pathogens or direct release through the formation of amphisomes [11] or exomeres [12] by cancer cells. Interestingly, it has been shown that during sepsis, megakaryocytes can produce histone‐rich platelets, which promote inflammatory and coagulation responses [13]. From all these processes, the most well known is neutrophil extracellular traps (NETs) formation, by which the nuclear content, including histones, is released from neutrophils upon stimulation during infection [6, 7, 14].

In recent years, various studies have described the role of circulating histones in the pathophysiology of infectious diseases, sepsis [15], ischemia–reperfusion processes [16], several cancers [17], multiple trauma [18, 19], COVID‐19 [20], acute lung injury [21], acute liver and lung failure [22], drug‐induced toxicity, and myocardial infarction, as reviewed by Chen et al. [3].

In this review, we aim to provide a comprehensive overview of the critical roles of extracellular histones in mediating inflammation, immune responses, and cell death. We explore their potential as biomarkers, emphasizing how their presence in blood drives pathological processes. Furthermore, we discuss how quantitative proteomics serves as a powerful tool for identifying and using circulating histones as biomarkers in severe conditions, offering insights into their diagnostic and prognostic value. Lastly, we delve into the potential use of circulating histones as biomarkers for treatment effectiveness monitoring.

### Highlights

- Extracellular histones mediate key processes in inflammation, immune responses, and cell death.
- Extracellular histones are potential biomarkers for diagnosis, prognosis, and treatment monitoring in critically ill conditions.
- Quantitative proteomics offers a promising tool for using circulating histones as biomarkers and for monitoring therapy effectiveness.

## Extracellular histones are key mediators in the pathophysiology of critical illnesses

### The role of extracellular histones in sepsis and septic shock

Sepsis is a syndrome caused by the dysregulation of the host's immune response to infection, which can rapidly progress to multi‐organ dysfunction syndrome and eventually lead to patient death if effective treatments are not applied immediately [23]. Septic shock is defined as a subset of sepsis in which underlying circulatory and cellular/metabolic abnormalities are profound enough to substantially increase mortality [24]. Approximately 2% of hospitalized patients and up to 75% of patients in intensive care units (ICUs) develop sepsis, of whom around 30% progress to septic shock. Sepsis cases have been rising by 7–9% each year. This trend is further exacerbated by the performance of multiple invasive procedures, the increase in bacterial resistance to antibiotics, the rise in immunosuppressive states, and the growing number of patients undergoing chemotherapy [25]. Despite advances in antibiotic treatments and supportive therapies, sepsis remains a significant global health challenge, with 48.9 million cases and approximately 11 million related deaths worldwide annually [23]. It has become the sixth leading cause of hospitalizations in the United States The mortality rate remains alarmingly high, highlighting the urgent need for early detection and effective management strategies to improve patient outcomes.

Scientific and clinical evidence has demonstrated that circulating histones participate as mediators in innate immunity by triggering inflammatory responses [26, 27], endothelium injury [28, 29], and the activation of the coagulation cascade [30], leading to coagulopathy [31, 32, 33], all closely related to the pathophysiology of sepsis and septic shock [34].

### The role of extracellular histones in SARS‐CoV2‐induced viral sepsis

Circulating histones are key mediators in the pathophysiology of severe COVID‐19, often referred to as “viral sepsis” by SARS‐CoV2 due to the shared clinical features between both severe conditions [35]. In fact, histones contribute to the cytokine storm, monocyte dysregulation, and complications such as acute respiratory distress syndrome (ARDS), vascular hyperpermeability, pulmonary inflammation, and thrombosis [35]. Elevated levels of NETs, which release circulating histones, have been found in the plasma and tracheal aspirates of critically ill patients with COVID‐19. Their role as signaling molecules and drivers of disease progression make them clinically relevant biomarkers and promising therapeutic targets for managing severe COVID‐19 [36], and probably other viral sepsis. Circulating histone levels at the time of hospital admission in COVID‐19 patients have been shown to be significantly elevated compared to controls and were particularly high in non‐survivors. Notably, these levels exhibit a strong positive correlation with D‐dimer, cardiac troponin, and fibrinogen, linking circulating histones to COVID‐19‐associated coagulopathy and indicating cardiac and endothelial tissue damage. Conversely, a negative correlation of circulating histones was observed with lymphocyte and other immune cell counts, suggesting that immune cell death is a major source of circulating histones in SARS‐CoV‐2 infection [37], although the effect of circulating histones initiating immune cell death should also be considered.

## Extracellular histones mediate cell cytotoxicity and contribute to multiorgan failure

Research has demonstrated that extracellular histones are pro‐inflammatory, pro‐coagulant, and cytotoxic [15, 38, 39]. This pleiotropic role of histones is due to histones acting as damage‐associated molecular patterns (DAMPs), activating innate immunity through toll‐like receptors (i.e., TLR2 and TLR4) and initiating multiple responses such as the inflammasome NLRP3‐dependent pathways [38], producing endothelial damage [28, 40], and interacting with several factors participating in the extrinsic and common pathway of coagulation [39, 41].

### Extracellular histones cause severe damage in endothelium

**Fig. 2:**
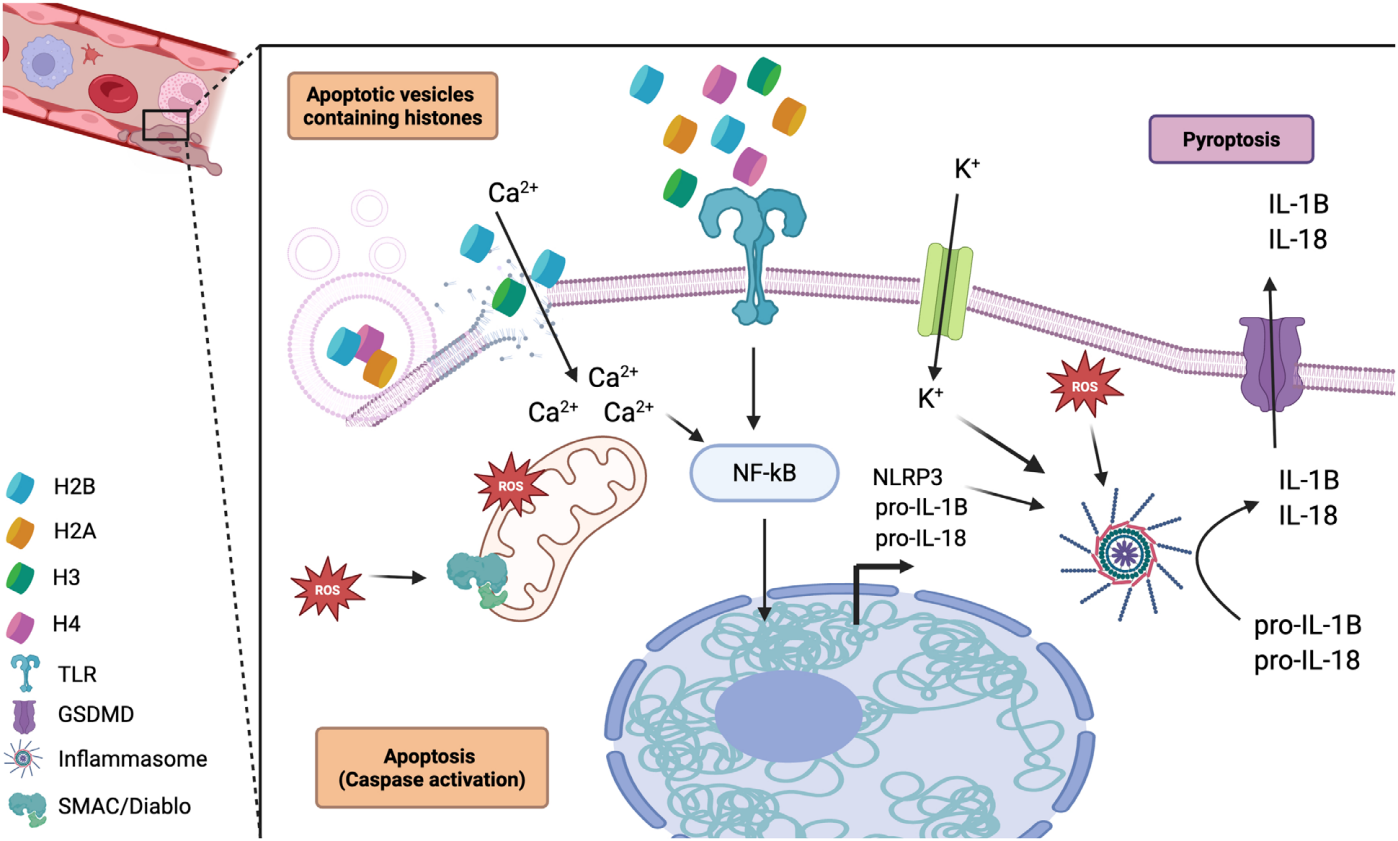
The pathophysiological roles of extracellular histones in the endothelium. Extracellular histones, released during cell death, act as damage‐associated molecular patterns (DAMPs) and play a pivotal role in mediating endothelial dysfunction. These histones bind to pattern recognition receptors, such as Toll‐like receptors TLR2 and TLR4, activating downstream signaling pathways including NF‐κB. This activation promotes the transcription of pro‐inflammatory cytokines and components of the NLRP3 inflammasome. Once NLRP3 is upregulated, cellular stressors such as potassium efflux (K^+^) and increased reactive oxygen species (ROS) trigger inflammasome oligomerization. This leads to the activation of caspase‐1 and the release of mature IL‐1β and IL‐18, key mediators of hyperinflammation, and induces pyroptosis. Concurrently, extracellular histones increase membrane permeability to calcium ions (Ca^2+^), which exacerbates mitochondrial dysfunction and further amplifies ROS production. ROS contribute to the activation of the Second Mitochondria‐derived Activator of Caspases (SMAD/Diablo), promoting the intrinsic apoptotic pathway through caspase activation. Moreover, internalized histones can directly disrupt the mitochondrial membrane potential, intensifying ROS generation and reinforcing both apoptotic and pyroptotic pathways. Together, these mechanisms highlight the multifaceted cytotoxic and proinflammatory roles of extracellular histones in endothelial injury. Created in BioRender. García‐Gimenez, J. (2025) Created in BioRender. García‐Gimenez, J. (2025) https://BioRender.com/wo4c8db

Extracellular histones are potent DAMPs that exert severe cytotoxic effects on endothelial cells, leading to vascular dysfunction and contributing to disease progression in conditions such as sepsis, thrombosis, and acute organ injury [28]. Exposure to circulating histones triggers multiple cell death pathways, including apoptosis, autophagy, and pyroptosis, thereby compromising endothelial integrity (Fig. 2). Histone‐induced apoptosis is mediated by mitochondrial dysfunction, increased Bax/Bcl‐2 ratio, and caspase activation, ultimately resulting in endothelial cell death [42]. Extracellular histones have a special affinity for membrane phospholipids, which leads to their integration into cell membranes and inducing significant inward ion currents [43]. This results in endothelial dysfunction, characterized by impaired endothelium‐dependent dilation and increased endothelial cell death [44]. Additionally, extracellular histones promote autophagy via the Sestrin2/AMPK/ULK1‐mTOR signaling pathway, leading to autophagic vesicle accumulation, which may initially serve as a protective response but ultimately contributes to endothelial dysfunction when excessively activated. Moreover, histones activate the NLRP3 inflammasome, triggering pyroptosis [27, 40, 45], an inflammatory form of programmed cell death that results in caspase‐1 activation, IL‐1β and IL‐18 secretion, and membrane rupture [27, 46]. The combined effects of these pathways lead to endothelial barrier disruption, increased vascular permeability, and heightened inflammation, all of which exacerbate tissue damage and contribute to disease severity [47]. Moreover, extracellular histones promote endothelial injury and coagulopathy by activating platelets and thrombin generation, and impairing anticoagulant pathways, leading to disseminated intravascular coagulation (DIC) [48]. This results in microvascular thrombosis and simultaneous hemorrhagic complications, worsening organ dysfunction. The interplay between histone‐induced endothelial damage, immune activation, and coagulation dysregulation creates a cycle that exacerbates sepsis‐associated coagulopathy [41, 49], fueling inflammasome activation, pyroptosis, and increased vascular permeability, ultimately intensifying systemic inflammation and disease progression [50].

### Extracellular histones induce myocardial dysfunction

In patients without prior cardiac disease, high histone levels have been associated with new‐onset left ventricular dysfunction and arrhythmias and predict worse outcomes when combined with elevated cardiac troponin [51]. *Ex vivo* studies showed that histones directly induce cardiomyocyte death through calcium overload. In septic mice, circulating histones caused cardiac injury, arrhythmias, and ventricular dysfunction [52]. Interestingly, cardiomyocyte death through ferroptosis is a key process in the pathophysiological basis of cardiovascular diseases [53]. Given that histone H3 has been shown to induce ferroptosis in endothelial cells [54] and macrophages [55], it is plausible that extracellular histones may mediate the activation of ferroptosis in cardiomyocytes.

Circulating histones have also shown their potential as diagnostic biomarkers for heart failure and its subtypes. In this regard, H2A levels were significantly higher in heart failure with reduced ejection fraction patients compared to reduced preserved fraction patients [56].

Sepsis‐induced cardiomyopathy (SIC) is a recognized but complex form of transient or reversible cardiac dysfunction observed in septic patients [57]. This condition is characterized by impaired myocardial contractility, reduced ejection fraction, and ventricular dilation, which can contribute to high mortality rates [58]. The pathophysiology of SIC involves systemic inflammation, oxidative stress, mitochondrial dysfunction, and dysregulated calcium homeostasis, which collectively lead to cardiomyocyte damage and apoptosis [59]. The presence of persistent inflammation in sepsis can destabilize atherosclerotic plaques, increasing the risk of myocardial infarction and stroke [60]. The role of circulating histones in cardiovascular diseases and cardiac injury is significant [51]. Specifically, circulating histones are released following an adverse cardiovascular event, such as ischemic heart disease [61], stroke [62], and coronary artery disease, in which an atherosclerotic plaque disruption occurs, with a subsequent intraluminal thrombus formation [61]. Moreover, the dose‐dependent effects of circulating histones on left ventricle and right ventricle function at clinically relevant concentrations in mouse models have demonstrated the impact of circulating histones in cardiac injury [52].

### Extracellular histones contribute to acute lung injury

Extracellular histones play a crucial role in the pathogenesis of ARDS by contributing to inflammation, cytotoxicity, and barrier dysfunction in the lungs. When histones are released into circulation, they can trigger excessive immune responses and increase endothelial and epithelial permeability, leading to lung injury [18]. The presence of extracellular histones in bronchoalveolar lavage fluid (BALF) and plasma of ARDS patients has been documented, with histone levels correlating with disease severity [63]. Specifically, high levels of circulating histones are associated with increased inflammatory cytokines (i.e., TNF‐α, IL‐6, IL‐1β), endothelial cell damage, and enhanced neutrophil infiltration in lungs, therefore contributing to alveolar–capillary barrier dysfunction, pulmonary edema, and impaired oxygenation [63].

### The role of extracellular histones in pancreatitis

Circulating histones and nucleosomes play a pivotal role in the pathogenesis and progression of acute pancreatitis (AP) and pancreatic cancer [64, 65]. Histones, released early during AP from necrotic acinar cells and activated immune cells, act as DAMPs that disrupt cell membranes and promote systemic inflammation. Once in the extracellular space, these histones bind to pattern recognition receptors such as TLR2 and TLR4 and activate, among other pathways, the inflammasome, leading to the release of proinflammatory cytokines, as described above and shown in Fig. 2. This cascade contributes to oxidative stress, immune cell recruitment, and amplification of the inflammatory response. Moreover, in the pancreas, extracellular histones compromise the integrity of the acinar cell plasma membrane, promoting necrosis and exacerbating local tissue damage [66]. Liu et al. showed that in patients with AP, circulating histone levels measured within 48 h of symptom onset had high predictive value for persistent organ failure and mortality [64]. In this regard, circulating histones damage pancreatic cells by directly causing necrosis through membrane disruption and by amplifying inflammatory responses, which together lead to worsening pancreatic injury and systemic complications in acute pancreatitis. Specifically, Szatmary et al. described how circulating histones released during acute pancreatitis accumulate in the pancreas and interact with pancreatic acinar cells. They induce charge‐ and concentration‐dependent leakage of the plasmalemma (cell membrane), leading to necrosis of these cells [67]. This effect is independent of extracellular calcium, indicating that histones themselves are directly cytotoxic to pancreatic cells, contributing to tissue damage in AP [67].

### Extracellular histones contribute to acute kidney injury

Sepsis‐induced kidney injury arises from a complex interplay of systemic inflammation, microcirculatory dysfunction, and direct cellular damage, with circulating histones further exacerbating the injury. The excessive production of pro‐inflammatory cytokines such as TNF‐α, IL‐1, and IL‐6 triggers endothelial activation, increases vascular permeability, and promotes leukocyte adhesion, ultimately compromising renal microcirculation [68]. This inflammatory cascade also induces oxidative stress and mitochondrial dysfunction, further aggravating renal injury. Additionally, microcirculatory dysfunction plays a key role in sepsis‐associated acute kidney injury (SA‐AKI), as alterations in vasoregulation, including imbalances between vasodilators like nitric oxide and vasoconstrictors such as endothelin‐1, lead to heterogeneous blood flow distribution and tissue hypoxia [68]. The degradation of the endothelial glycocalyx further compromises microvascular integrity, exacerbating perfusion deficits and cellular damage [69]. Consequently, renal ischemia and hypoxia activate apoptosis and necroptosis pathways in tubular cells, leading to impaired kidney function. Additionally, apoptosis and necroptosis contribute to the release of extracellular histones, further escalating tissue damage and inflammation. In fact, Nakazawa et al. investigated the role of extracellular histones and NETs in acute kidney injury (AKI) and found that ischemic kidney injury leads to the release of histones from necrotic tubular epithelial cells, which in turn prime neutrophils to form NETs. These NETs and associated histones exacerbate tubular cell death and promote further NET formation, creating a detrimental feedback loop [70]. Also, it has been demonstrated that warm ischemia (28 °C) leads to significantly higher histone H3 release from renal cells than cold ischemia (4 °C). In fact, histones were found in cytoplasmic protrusions and extracellular debris of damaged glomerular cells, contributing to renal injury and inflammation [71].

Additional evidence demonstrated the important role of extracellular histones in renal damage by showing that histone neutralization with an anti‐histone antibody effectively reduced postischemic liver and kidney damage [72]. Similarly, Wang et al. reported that heparin treatment in a murine sepsis model significantly reduced histone‐mediated kidney injury by lowering histone concentrations, inflammatory markers, and tissue damage [73]. Additionally, Reutelingsperger et al. demonstrated that M6229, a low‐anticoagulant heparin fraction, protected against histone‐induced liver injury, kidney dysfunction, and mortality in a rat model of acute hyperinflammation, supporting the therapeutic potential of histone‐neutralizing agents [74].

## Circulating histones are potential biomarkers for critically ill patient management

Excessive neutrophil activation leads to the release of NETs [75, 76], increasing the levels of extracellular histones and nucleosomes. These, in turn, amplify the cytotoxic effects by promoting inflammation, immune thrombosis [39, 77], and organ damage [22, 38]. This cascade of events exacerbates disease progression and severity, further compromising patient outcomes.

### Blood circulating histones as biomarkers for conditions of severe illness

The diagnosis of sepsis and septic shock can be challenging due to the nonspecific symptoms that often appear in the early stages, especially in patients with complex pre‐existing conditions [34, 78]. Additionally, many of these patients receive antimicrobial therapies that can result in negative microbiological culture results. In fact, between 30% and 40% of patients with sepsis in ICUs have negative cultures [78]. Even when cultures are positive, the results can take days to become available, delaying the diagnosis, complicating clinical decision‐making, and reducing survival chances. Therefore, the diagnosis of sepsis relies on the evaluation of clinical signs (such as fever, tachycardia, and tachypnoea) along with microbiological data if available, leukocyte count, and nonspecific biochemical biomarkers like lactate, which in sepsis generally shows elevated levels above established thresholds. The current consensus approach for sepsis and septic shock diagnosis is defined by Sepsis‐3 consensus [24]. While this approach is useful, it has limitations in terms of precision and speed, particularly in critical care settings.

Considerable efforts have been made to identify new diagnostic and predictive biological markers that are both sensitive and specific [79]. Among these, C‐reactive protein (CRP), procalcitonin (PCT), and proadrenomedullin (PADM) have emerged as notable candidates [80, 81, 82, 83], although none of them has proven to be sufficient for the management of a severe and heterogeneous condition such as sepsis [84].

Circulating histones are gaining increased attention as biomarkers for the diagnosis and prognosis of sepsis. In 2009, Xu et al. proposed the possibility of using histones as diagnostic and prognostic markers for sepsis, as well as the potential therapeutic application of histone antagonists, such as specific antibodies, to prevent death from sepsis [15]. Continuous research has shown that circulating histone levels are significantly elevated in sepsis patients compared to control individuals (without associated septic processes and hospitalized in ICUs) [22, 51, 85, 86, 87]. A summary of the most relevant information from studies using circulating histones and nucleosomes as biomarkers is shown in Table 1.

**Table 1:** Circulating histones as biomarkers in critical illness: cutoff levels, performance, pathophysiological roles, and detection methods. In this table, we have only included studies quantifying circulating histones in international units (μg·mL^−1^) and not arbitrary units. AUC, area under the curve; cTn, cardiac troponin; CRRT, continuous renal replacement therapy; DIC, disseminated intravascular coagulation; N.A., not available; SIRS, systemic inflammatory response syndrome; SOFA, sequential organ failure assessment; Sn, sensitivity; Sp, specificity.

Histones	Levels (μg·mL^−1^)	Sn/Sp AUC	Use of the biomarker	Method of detection	Explanation of the study	Reference
Sepsis and septic shock						
Circulating H3	H3 levels 0.011 [0.002–0.048] μg·mL^−1^) were significantly higher than those in patients without coagulation failure (0.035 [0.015–0.059] μg·mL^−1^) H3 > 0.02 μg·mL^−1^ were associated to high DIC score	For prediction of 28‐day mortality with an AUC 0.73	Prognostic	ELISA	85 patients with suspected infections and SOFA score of ≥2. 40 patients (47.1%) were associated with shock and 12 patients (14.1%) died by day 28.	[31]
Circulating H3	H3 (μg·mL^−1^) 0.5 [0–8.3] sepsis 0.6 [0.4–2.9] severe sepsis 3.1 [0.3–5.8] septic shock 3.15 μg·mL^−1^ in non‐surviving septic patients versus 0.57 μg·mL^−1^ in septic‐surviving patients (*P* = 0.040)	N.A.	Diagnostic and Prognostic	Immunoassay based on western blot	Plasma from 43 septic patients who were admitted to the ICU	[88]
Circulating H2B and H3	H2B > 10.1 μg·mL^−1^ (95%CI: 2076.16–18137.62) and H3 > 20.09 μg·mL^−1^ (95%CI: 5,74–34.23) were associated to poor outcome in septic shock patients (*P* < 0.05)	For prediction of mortality using cutoff point of H3 7.56 μg·mL^−1^, Sn 75%, Sp 88.9% and AUC 0.806	Prognostic	Mass spectrometry‐based method	Plasma samples from 17 septic shock patients and 10 controls. Septic shock patients were divided in survivors (*n* = 9) and non‐survivors (*n* = 8) in an ICU environment	[85]
Circulating H2B and H3	H2B > 0.214 μg·mL^−1^ were indicative of Septic Shock H2B > 0.914 μg·mL^−1^ and H3 > 0.370 μg·mL^−1^) associated with more severe cases with fatal outcome (*P* < 0.05)	For prediction of mortality using cutoff point of H3 0.48 μg·mL^−1^, Sn 66.7%, Sp 73.9% and AUC 0.720	Diagnostic and prognostic	Mass spectrometry‐based method	Plasma samples from critically ill patients without sepsis (*n* = 9), septic patients (*n* = 11) and septic shock (*n* = 69). Septic shock patients were divided in survivors (*n* = 56) and non‐survivors (*n* = 13)	[86]
Circulating histones	Circulating histones were significantly elevated to 63.5 μg·mL^−1^ [22.2, 78.3] compared with normal controls 1.5 μg·mL^−1^ [0.7, 2.1] (*P* < 0.05)	For prediction of 28‐day mortality in patients with sepsis using a histone cutoff value of 75 μg·mL^−1^ AUC 0.744 (*P* = 0.003) Sn 60%, Sp 86.1%	Prognostic, Circulating Histones and cTn correlate with sepsis severity and outcome	ELISA Cell Death Detection ELISAPLUS kit (Roche Diagnostics)	Plasma samples from 65 patients with sepsis, and from 27 healthy controls	[51]
Circulating nucleosomes	SIRS without infection 1.1 ± 1.7 μg·mL^−1^; Sepsis 1.7 ± 1.9 μg·mL^−1^ (*P* = 0.036); Severe sepsis 3.0 ± 9.4 μg·mL^−1^; septic shock 5.5 ± 10.9 μg·mL^−1^ (*P* = 0.012) And difference between survivors and non‐survivors (*P* = 0.007)	AUC 0.63 (0.53–0.73) to distinguish sepsis from SIRS AUC 0.75 (0.62–0.87) for predicting 28‐day mortality	Diagnostic and prognostic	ELISA Cell Death Detection ELISAPLUS kit (Roche Diagnostics)	Plasma samples from 24 non‐infectious SIRS; 4 uncomplicated sepsis; 127 severe sepsis; and 35 septic shock. Patient Outcome: 190 survivors and 13 non‐survivors	[93]
Circulating H4	H4 > 0.300 ± 0.150 μg·mL^−1^ in septic patients (compared to 0.090 ± 0.020 μg·mL^−1^ in control subjects) (*P* < 0.001) Patients with plasma histone levels ≥0.300 μg·mL^−1^ experienced markedly worse clinical outcomes than those with levels <0.300 μg·mL^−1^ (*P* < 0.0001)	For prediction of mortality using cutoff point 0.300 μg·mL^−1^, Sn 70.3%, Sp 76.4% and AUC 0.731.	Prognostic	Immunoassay	Plasma samples from septic patients (*n* = 136) and healthy controls (*n* = 10). Septic patients were divided in survivors (*n* = 89) and non‐survivors (*n* = 37)	[90]
Circulating nucleosomes (Nu.H3.1)	Healthy donor's median [0.015 μg·mL^−1^]. Higher levels of H3.1 in septic shock patients. Levels peaked at Day 1–2 (1.515 μg·mL^−1^) and then declined progressively during the following days. Higher levels of H3.1 in non‐survivors 1.919 (0.880–12.098 μg·mL^−1^) compared to survivors 1.333 (0.385–3.637) μg·mL^−1^ (*P* < 0.0001)	For prediction of 28‐day and 5‐day mortality using cutoff point 4.63 μg·mL^−1^ AUC 0.63 and AUC 0.66, respectively	Prognostic	NuQ H3.1® assay (Volition)	Plasma samples from 151 patients (with samples taken at different time points after admission)	[92]
Circulating nucleosomes (Nu.H3.1)	H3.1 median levels of 0.443 μg·mL^−1^ in sepsis and median 0.921 μg·mL^−1^ in septic shock. H3.1 was also higher in patients CRRT with septic shock vs sepsis (1.832 μg·mL^−1^ vs 0.801 μg·mL^−1^, *P* = 0.01)	H3.1 levels demonstrated moderate discriminatory performance for RRT decision. AUC of 0703% (95% CI 0.661–0.746)	Diagnostic and prediction of CRRT	NuQ H3.1^®^ assay (Volition)	971 critically ill patients: 443 patients with sepsis, and 520 with septic shock and 8 patients with unknow diagnosis according to Sepsis‐3	[91]
COVID‐19						
Circulating histones	Mild; 2.00 μg·mL^−1^ [0.68–6.62], Severe; 9.75 μg·mL^−1^ [3.61–21.88], Critical; 23.37 μg·mL^−1^ [11.35–30.02] (*P* < 0.001) Patients with elevated histones required critical care admission (*P* < 0.001), increased duration of mechanical ventilation (R = 0.778, *P* = 0.022) and overall length of hospital stay (R = 0.618, *P* < 0.001)	N.A.	Prognostic and therapy management	N.A.	Plasma samples from COVID‐19 patients categorized based on disease severity into four groups: mild (*n* = 30), moderate (*n* = 38), critical (*n* = 20), and non‐survivors (*n* = 25)	[49]
Circulating histones	Healthy controls, median = 2.9 μg·mL^−1^ [IQR = 1.5–3.3]; mild, 2.6 μg·mL^−1^ [0.7–7.6]; moderate, 10.5 μg·mL^−1^ [3.5–27.2]; critical, 20.0 μg·mL^−1^ [6.2–33.0]; non‐survivors, 29.6 μg·mL^−1^ [11.2–60.0] Circulating histone levels were markedly higher in non‐survivors (29.6 μg·mL^−1^ [11.2–60.0] compared to survivors (8.6 μg·mL^−1^ [3.1–24.8], *P* = 0.002)	N.A.	Diagnostic and prognostic, high histone levels correlated with D‐dimer	In‐house and validated ELISA using a monoclonal anti‐histone H3 (citrulline R8) antibody (Abcam) for capture and a monoclonal anti‐DNA antibody (Cell Death ELISAPLUS, Roche) for detection	Plasma samples from 113 COVID‐19‐associated coagulopathy patients	[37]
Circulating nucleosomes (Nu.H3.1)	Nucleosome Nu.H3.1 levels were markedly higher in non‐survivors 1.77 [0.439–15.848] μg·mL^−1^ compared to survivors 1.08 [0.222–4.295] 6 μg·mL^−1^ *P* = 0.098)	N.A.	Prognostic	NuQ H3.1^®^ assay (Volition)	Plasma samples from 48 control subjects, 46 septic shock patients and 22 critically ill patients with COVID‐19	[96]

Yokoyama et al. measured circulating histone H3 levels using ELISA in septic patients and explored their association with coagulation failure, organ dysfunction, and mortality. The study included 85 patients with suspected infections and a sepsis‐related organ failure assessment (SOFA) score ≥2, among whom 47.1% developed septic shock, and 14.1% died within 28 days. The authors reported that histone H3 levels were significantly higher in patients with coagulation failure (median: 0.035 μg·mL^−1^, IQR: 0.015–0.059) compared to those without (median: 0.011 μg·mL^−1^, IQR: 0.002–0.048). Additionally, histone H3 levels exceeding 0.02 μg·mL^−1^ were associated with an increased disseminated intravascular coagulation (DIC) score. The biomarker demonstrated a prognostic utility for predicting 28‐day mortality, with an area under the curve (AUC) of 0.73 [31].

Other approaches have used semiquantitative immunoassay methods based on western blot. Wildhagen et al. measured histone H3 levels in plasma samples from 43 ICU‐admitted sepsis patients and found a correlation of increased H3 levels with disease severity: 0.5 μg·mL^−1^ [0–8.3] in sepsis, 0.6 μg·mL^−1^ [0.4–2.9] in severe sepsis, and 3.1 μg·mL^−1^ [0.3–5.8] in septic shock. Notably, non‐surviving septic patients had significantly higher H3 levels (3.15 μg·mL^−1^) compared to survivors (0.57 μg·mL^−1^) (*P* = 0.040) [88].

We have used a different approach based on mass spectrometry to quantify circulating histones levels in plasma samples. Elevated H2B (>10.1 μg·mL^−1^) and H3 (>20.09 μg·mL^−1^) levels were linked to poor prognosis in septic shock patients [85]. We also compared the levels of circulating histones among patients admitted to the ICU and found elevated H2B levels above 0.121 μg·mL^−1^ to be indicative of septic shock, with higher circulating histone levels (H2B > 0.435 μg·mL^−1^ and H3 > 0.300 μg·mL^−1^) associated with more severe cases involving organ failure and requiring invasive support. Patients with DIC exhibited H2B and H3 levels above 0.040 μg·mL^−1^ and 0.258 μg·mL^−1^, respectively [86]. In this regard, Ito et al. also proposed a new scoring system using serum histone H3 levels and platelet counts to predict coagulopathy in septic patients. Values above 0.09 μg·mL^−1^ of circulating H3 were indicative of DIC [89].

Lu et al. investigated the prognostic value of plasma histone H4 levels in sepsis patients. The researchers measured plasma histone H4 concentrations in 136 septic patients and 10 healthy controls using an immunoassay. They found that septic patients had significantly higher histone H4 levels (0.300 ± 0.150 μg·mL^−1^) compared to controls (0.090 ± 0.020 μg·mL^−1^). A cutoff value of 0.300 μg·mL^−1^ for histone H4 predicted mortality with a sensitivity of 70.3%, specificity of 76.4%, and an AUC of 0.731. Patients with histone H4 levels ≥0.300 μg·mL^−1^ experienced markedly worse clinical outcomes than those with lower levels. The study concluded that elevated plasma histone H4 is a significant prognostic biomarker in sepsis [90].

In the SISPCT randomized clinical trial, Neumann et al. found that at patient admission (971 critically ill patients) H3.1 nucleosome levels were significantly higher in patients with septic shock compared to those with sepsis alone (median 0.921 vs. 0.432 μg·mL^−1^, *P* < 0.001), suggesting a potential role as a diagnostic and prognostic marker at ICU admission. Additionally, higher H3.1 levels were independently associated with increased mortality risk and a greater likelihood of continuous renal replacement therapy (CRRT). Patients who required CRRT had significantly elevated nucleosome H3.1 levels, particularly those with septic shock (median 1.832 μg·mL^−1^ vs. 0.8014 μg·mL^−1^ in sepsis, *P* = 0.01). The biomarker demonstrated moderate discriminatory ability for CRRT decision‐making, with an AUC of 0.738 (95% CI: 0.661–0.746). [91].

Rahimi's study investigated the prognostic value of circulating nucleosomes, using the Nu.H3.1 test, in plasma samples from 151 septic shock patients. Higher levels of NuH3.1 were observed in non‐survivors compared to survivors, with median values of 1.91 μg·mL^−1^ (0.88–12.09 μg·mL^−1^) in non‐survivors versus 1.33 μg·mL^−1^ (0.385–3.637 μg·mL^−1^) in survivors. The levels peaked between Day 1–2 (1.51 μg·mL^−1^) and declined progressively in the following days. The study also established a cutoff value of 4.63 μg·mL^−1^ for predicting 28‐day mortality (AUC = 0.63) and 5‐day mortality (AUC = 0.66) [92].

Duplessis et al. investigated the prognostic and diagnostic utility of circulating nucleosomes in sepsis by measuring their plasma levels in patients with systemic inflammatory response syndrome (SIRS), sepsis, severe sepsis, and septic shock using the Cell Death Detection ELISAPLUS kit (Roche Diagnostics). The study demonstrated that nucleosome levels increased with disease severity, with median concentrations of 1.1 ± 1.7 μg·mL^−1^ in SIRS, 1.7 ± 1.9 μg·mL^−1^ in sepsis, 3.0 ± 9.4 μg·mL^−1^ in severe sepsis, and 5.5 ± 10.9 μg·mL^−1^ in septic shock. The biomarker exhibited moderate discriminative capacity, with an AUC of 0.63 (95% CI: 0.53–0.73) for differentiating sepsis from SIRS and an AUC of 0.75 (95% CI: 0.62–0.87) for predicting 28‐day mortality [93].

Elevated levels of histone H4 have also been found in patients with organ failure compared to those with minor trauma [94]. Moreover, histone H4 levels, determined by immunoassay, were significantly elevated in septic patients, averaging 0.300 ± 0.150 μg·mL^−1^, compared to 0.090 ± 0.020 μg·mL^−1^ in control subjects. Particularly, patients with plasma histone levels ≥0.300 μg·mL^−1^ experienced markedly worse clinical outcomes than those with levels <0.300 μg·mL^−1^. These individuals had higher SOFA scores, increased mortality rates, greater reliance on vasopressor support, and elevated plasma levels of cardiac troponin (TnI), NT‐proBNP, lactate, and PCT, reflecting heightened disease severity and systemic dysfunction [90]. Elevated levels of circulating histones were found in ARDS patients, with histone concentrations exceeding 50 μg·mL^−1^ in patient sera being cytotoxic to endothelial cells, while a cutoff value of 75 μg·mL^−1^ was predictive of increased ICU mortality [63]. Importantly, high levels of circulating histones were indicative of cardiac injury; 75% of patients (9/12) with levels higher than 75 μg·mL^−1^ developed left ventricular dysfunction compared to 8.3% of patients (2/24) (*P* < 0.001) with histones less than 75 μg·mL^−1^ [51].

These findings suggest that circulating histones may serve as valuable diagnostic and prognostic biomarkers in sepsis, aiding in early identification, disease severity stratification, and assessment of organ failure.

### Blood circulating histones as biomarkers in viral sepsis induced by SARS‐CoV2 infection

Viode *et al*. developed a high‐throughput liquid chromatography–mass spectrometry (LC–MS)–based method to analyze plasma samples from 1117 SARS‐CoV‐2‐infected patients over 28 days, quantifying 2910 plasma circulating proteins [95]. Among the identified biomarkers, high levels of AHNAK and H2A clustered histone 20 (H2AC20) at admission were associated with the need for extracorporeal membrane oxygenation and/or invasive mechanical ventilation, while elevated histone H1.5 during the first week after admission was linked to fatal outcomes [95]. Shaw *et al*. showed that patients with circulating histone levels above the cytotoxic threshold of 30 μg·mL^−1^ exhibited significantly worse clinical and laboratory parameters compared to those below the threshold. These patients had elevated levels of D‐dimer, fibrinogen, IL‐6, and CRP, along with reduced oxygen saturation (SpO_2_), a higher likelihood of requiring critical care admission, longer durations of mechanical ventilation (*R* = 0.635), and extended hospital stays (*R* = 0.654). Circulating histone levels were significantly (*P* < 0.001) elevated in patients with increasing severity of COVID‐19 infection (Mild; 2.00 μg·mL^−1^ [0.68–6.62], Severe; 9.75 μg·mL^−1^ [3.61–21.88], Critical; 23.37 μg·mL^−1^ [11.35–30.02]) [49]. Another study developed by Shaw et al. also evaluated the levels of circulating nucleosomes in 113 patients with COVID‐19, measured by an in‐house manufactured kit, as a COVID‐19‐associated coagulopathy and mortality biomarker. The findings revealed that circulating histone levels were significantly elevated in critically ill COVID‐19 patients, with a median of 20.0 μg·mL^−1^ (IQR: 6.2–33.0) in critical cases, compared to 2.9 μg·mL^−1^ (IQR: 1.5–3.3) in healthy controls. Furthermore, histone levels were markedly higher in non‐survivors (29.6 μg·mL^−1^, IQR: 11.2–60.0) compared to survivors (8.6 μg·mL^−1^, IQR: 3.1–24.8, *P* = 0.002), suggesting their potential role as prognostic markers. Importantly, histone levels correlated with D‐dimer and were associated with lower platelet counts, prolonged prothrombin time, and reduced antithrombin levels (*P* = 0.048), parameters linked to a disseminated intravascular coagulation phenotype [37].

Moreover, the study by Morimont et al. evaluated circulating nucleosomes (Nu.H3.1) in 48 control subjects, 46 septic shock patients, and 22 critically ill patients with COVID‐19, observing a trend toward higher Nu.H3.1 levels in non‐survivors (1.77 [0.43–15.84] μg·mL^−1^) compared to survivors (1.08 [0.22–4.29] μg·mL^−1^) [96].

Currently, there is limited literature directly comparing the performance of circulating histones with traditional biomarkers such as CRP and PCT. However, available data highlight the potential of histones as superior early indicators in critical care settings. In patients with AP, circulating histones measured within the first 24–48 h demonstrated high diagnostic accuracy for predicting persistent organ failure. At a cutoff value of ≥5.4 μg·mL^−1^, histones showed a sensitivity of 82.6%, specificity of 94.4%, and an AUC of 0.92 for organ failure. For mortality prediction, sensitivity reached 88.9% and specificity was 89.9% [64]. In contrast, CRP measured at 48 h (cutoff ≥250 mg/L) had similar sensitivity (80.0%) but lower specificity (77.1–80.5%) and markedly lower AUC values (0.54–0.61) for organ failure. Similarly, Szatmary et al. reported that histones (measured at the same cutoff of ≥5.4 μg·mL^−1^) achieved an AUC of 0.87, confirming their superiority over CRP in early disease stratification [67]. Penttilä et al. found that nucleosomes outperformed CRP in patients without initial organ dysfunction (AUC 0.648 vs. 0.539) and were the only independent predictor of progression to severe disease [97]. Altogether, these findings emphasize the dual role of extracellular histones and nucleosomes as early biomarkers and mediators of injury in AP, offering novel opportunities for risk assessment and therapeutic targeting.

## Quantitative proteomic tools could measure circulating histones as biomarkers of severe conditions

Currently, only research‐use‐only (RUO) assays are available for detecting circulating histones or nucleosomes in blood samples. Among these, the ELISA Cell Death Detection ELISAPLUS kit (Roche Diagnostics) is the most widely used in research studies. Other commercially available tests include the ORG711 Nucleo‐9‐Line in a test strip format and the ELISA ORG528 kit, both marketed by Orgentec Diagnostika GmbH (Mainz, Germany). However, these kits are designed for the immunological detection of nucleosomes rather than specifically targeting histones. Another ELISA‐based kit available for research use is the Nu.Q™ Total Assay Test (Belgian Volition SRL, Isnes, Belgium). Recent studies have proposed the Nu.Q H3.1® Immunoassay as a promising tool for quantifying NETs by measuring histone H3.1 nucleosomes. This assay has gained particular interest in sepsis research and prognosis, as it enables the detection of circulating nucleosomes containing histone H3.1, providing valuable insights into disease severity. As discussed above and summarized in Table 1, measuring circulating H3.1 nucleosome levels could support early risk stratification and targeted interventions in critically ill patients. Moreover, it has been proposed as an independent predictor of 28‐day mortality and the need for CRRT in sepsis patients.

Beyond assays designed to measure total and H3.1 nucleosome detection, there are also ELISA kits designed to detect modified circulating histones, which play a crucial role in various pathological conditions, including sepsis. In this regard, EpiGentek offers several RUO kits for detecting modifications in H3 and H4 histones. Among these, the EpiQuik Circulating Modified Histone H3 Multiplex Assay Kit allows the simultaneous detection and quantification of 22 different posttranslational modifications (PTMs) of histone H3. These modifications are known to influence chromatin structure and immune responses, highlighting their potential significance in disease monitoring and prognostic assessment.

Mass spectrometry (MS) has emerged as a powerful tool for quantifying circulating histones in complex biological matrices, such as plasma, with high sensitivity and specificity. Among the available MS‐based approaches, liquid chromatography coupled with tandem mass spectrometry (LC–MS/MS) provides accurate absolute quantification of histones and their PTMs even in the presence of interfering plasma proteins. Additionally, the use of stable isotope‐labeled peptides allows for precise quantification through targeted MS approaches, such as parallel reaction monitoring (PRM) or selected reaction monitoring (SRM) [98]. In this regard, companies such as MRMproteomics Inc. (Montreal, Canada) and Promise (France) are developing assays for RUO and IVD assays to quantify circulating proteins in plasma. MRMproteomics Inc. is commercializing a kit (PeptiQuantTM Kit) for MRM‐MS analysis of 125 human plasma proteins (https://mrmproteomics.com/product/bak‐sc6500‐125/), but not detecting circulating histones. The company Promise Advanced Proteomics (Grenoble, France) is commercializing a mAbXmise Oncology kit (CE‐IVD) for the quantitation of therapeutic antibodies (Trastuzumab, Rituximab, Bevacizumab, Cetuximab, Nivolumab, Pembrolizumab, and Ipilimumab based on MRM‐MS method), but they have not yet developed a test to quantify circulating histones. Based on this technology and previously published work [85, 86], the company EpiDisease S.L (Valencia, Spain) is validating in a multicentric clinical assay the HistShock test to quantify circulating histones H3 and H2B [85, 86].

**Fig. 3:**
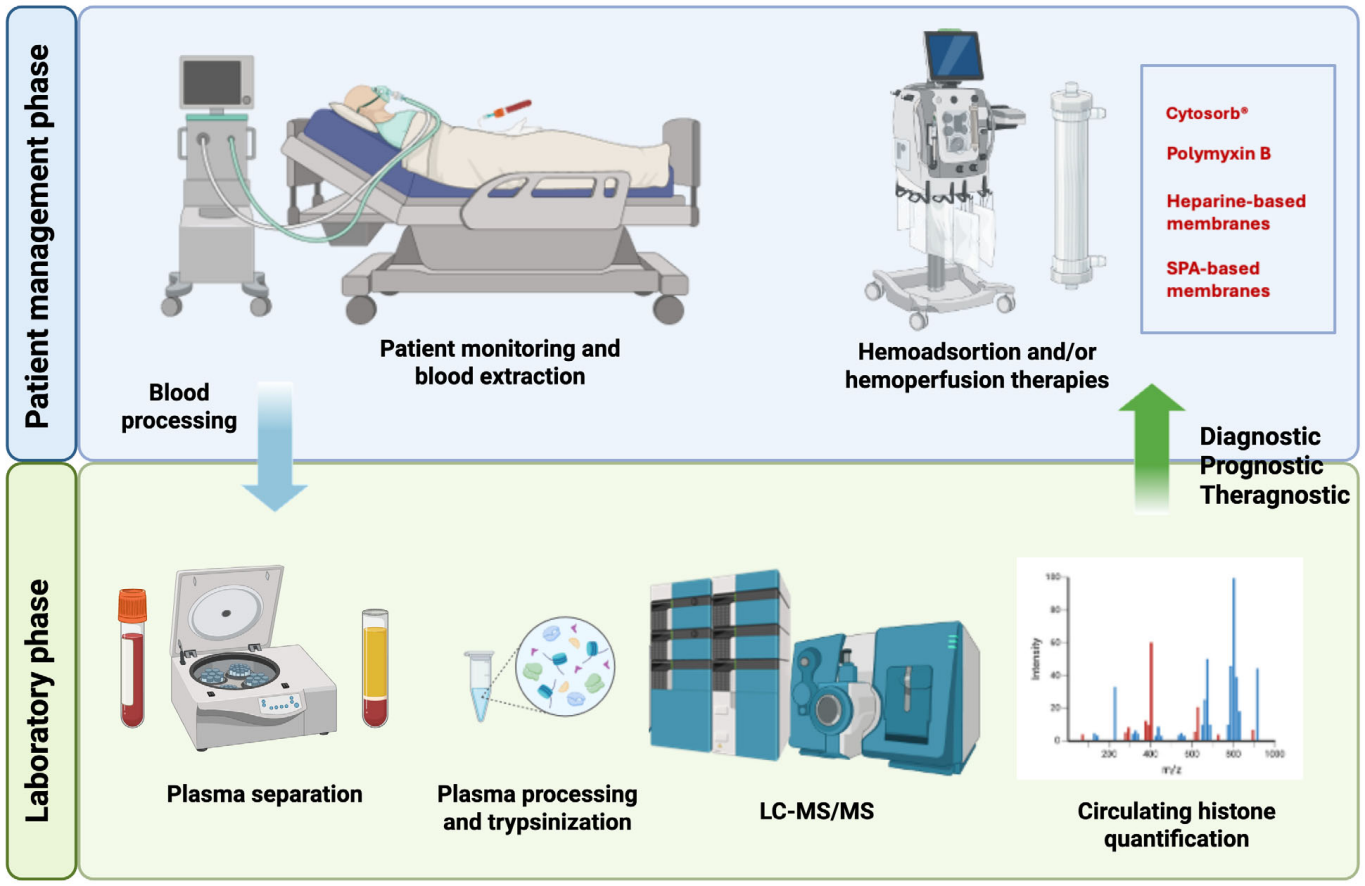
Integrating circulating histone measurements by MS for sepsis diagnosis, prognosis, and patient management with extracorporeal therapies. This figure presents a comprehensive approach to using quantitative mass spectrometry (MS) for personalized sepsis management. The workflow consists of two interconnected phases: (1) the patient management phase, where critically ill patients undergo blood extraction, clinical monitoring, and potential treatment with extracorporeal hemoadsorption or hemoperfusion therapies (e.g., CytoSorb®, Polymyxin B, heparin‐based membranes, and SPA‐based membranes) to remove circulating histones and other hyperinflammatory mediators; and (2) the laboratory phase, where plasma separation, processing, and LC–MS/MS analysis enable the quantification of circulating histones, providing diagnostic, prognostic, and theragnostic insights to advance personalized medicine in sepsis care. Created in BioRender. García‐Gimenez, J. (2025) https://BioRender.com/piejpbl

Recent advances in high‐resolution MS have enabled the simultaneous identification and quantification of circulating histones, as well as multiple PTMs, shedding light on their functional implications in pathology. These findings highlight the potential of MS as a quantitative tool for histone‐based biomarkers, facilitating early disease detection, prognostic, and personalized therapeutic intervention (Fig. 3).

## Potential clinical implications of circulating histones as targets in septic patient management

Circulating histone removal is becoming a promising strategy to develop personalized therapies in sepsis [99] (Fig. 3). However, due to their structural similarity to normal blood proteins, low abundance, and interference from other blood biomacromolecules, clearing histones from the bloodstream has been challenging. Several efforts have emerged in recent years. Extracorporeal blood purification offers a promising approach to eliminate circulating histones. Inspired by the natural electrostatic interactions between negatively charged DNA and positively charged histones, Chen et al. developed heparin‐mimicking hydrogel microspheres (RCHMs) as an innovative solution. These microspheres contain carboxyl and sulphonic acid groups, providing excellent hemocompatibility and the ability to adsorb histones via electrostatic interactions. The RCHMs demonstrated minimal hemolysis, blood cell activation, and complement activation, while efficiently adsorbing 91.16% of histones with a capacity of 20.47 μg·mg^−1^ within 4 h. Additionally, RCHMs attenuated histone‐induced thrombocytopenia, platelet aggregation, and endothelial cell death [100]. Another strategy to remove extracellular histones is based on the use of M6229, a low‐anticoagulant fraction of heparin (UFH) isolated using antithrombin‐based affinity chromatography, which effectively protected a rat model of acute hyperinflammation from histone‐induced liver damage, kidney dysfunction, and mortality [74]. Other strategies in development for neutralizing extracellular histones are based on the use of small polyanions (SPAs). Due to their cationic nature, histones interact with cell membranes, leading to cytotoxicity and coagulation activation. SPAs (~0.9–1.4 kDa) can electrostatically bind histones, reducing their harmful effects, including platelet activation and erythrocyte fragility in mice models [101]. In another study, Li and colleagues presented a novel approach for the selective removal of circulating histones [102]. Using phage display screening, two dodecapeptides, P1 (HNHHQLALVESY) and P2 (QMSMDLFGSNFV), were identified for their strong affinity to circulating histones while exhibiting negligible interactions with common blood proteins like human serum albumin, immunoglobulin G, and transferrin. The P1 peptide was integrated into a polymer matrix, poly(PEGMA‐co‐P1), immobilized onto a silica gel surface and efficiently captured histones from model solutions and whole blood while demonstrating excellent blood compatibility, antifouling properties, and resistance to interference [102].

The previous findings highlight the potential of certain hemoperfusion adsorbents as effective strategies for removing circulating histones in critically ill patients, offering a novel therapeutic approach for managing histone‐related pathological complications. While some circulating histones and other DAMPs are still undergoing validation, existing extracorporeal blood purification techniques for histone removal require further clinical evaluation. In this regard, CytoSorb® hemoadsorption and polymyxin B hemoperfusion have emerged as promising blood purification strategies. CytoSorb®, a broad‐spectrum hemoadsorption cartridge, showed its efficacy in removing DAMPs, including histones and HMGB1, in blood samples of 22 multiple injured patients collected at admission [103]. Studies suggest that CytoSorb® therapy can mitigate histone‐induced cardiotoxicity and endothelial dysfunction, potentially benefiting patients with sepsis and trauma. However, current evidence does not conclusively support the widespread use of CytoSorb® in intensive care medicine due to a lack of demonstrated mortality benefits across various conditions [104]. On the other hand, polymyxin B‐immobilized hemoperfusion seems to be effective in binding and removing pathogen‐associated molecular patterns (PAMPs) such as endotoxins [105], reducing endotoxin‐driven immune responses in sepsis patients [106], proving useful as precision medicine in patients with refractory septic shock, severe multiorgan dysfunction, and endotoxemia [107, 108, 109]. Finally, Seraph‐100 has demonstrated broad‐spectrum pathogen elimination, including drug‐resistant bacteria and viruses, with removal rates ranging from 30% to 99.9% per blood pass. In addition to pathogens, it binds DAMPs such as histones, HMGB1, and LPS‐binding protein [110], reducing inflammatory and renal injury in sepsis models. Clinical studies, particularly in COVID‐19 patients, suggest that early Seraph‐100 treatment improves survival, reducing SARS‐CoV‐2 viremia and inflammatory mediators, including those released by inflammasomes [110]. To date, there is no direct experimental evidence demonstrating the ability of Oxiris® or Jafron HA‐series hemoadsorption devices to remove extracellular histones or other DAMPs, such as histones, highlighting the need for further studies to evaluate their potential role in targeting these proinflammatory mediators.

In general, sequential biomarker monitoring during ICU stays remains limited, although some evidence suggests that baseline data at admission may help predict patient outcomes in the following days. Importantly, while most studies to date have focused on cross‐sectional comparisons of histone levels between clinical groups, emerging evidence underscores the potential value of serial measurements of circulating histones. For example, as described before, Viode et al. tracked histone levels over 28 days in hospitalized COVID‐19 patients and found that specific histone isoforms, such as H1.5, increased during the first week in non‐survivors, indicating potential for early risk stratification and outcome prediction [95]. Similarly, data from the NuQ H3.1® assay showed that nucleosome levels peaked at day 1–2 and declined over time, with persistent elevations associated with poor outcomes [92]. These findings underscore the unmet need for systematic evaluation of the kinetics of circulating histones and their potential role as dynamic biomarkers for treatment monitoring in sepsis and related critical conditions.

## Conclusions and perspectives

The elevated levels of extracellular histones in severe pathologies point out their role in cellular and tissue damage [15]. Therefore, circulating histones in biological fluids represent a potential biomarker for disease progression risk and prognosis. However, before the use of circulating histones as biomarkers, the method of measuring histones needs to be standardized, and large clinical trials to validate their use as clinical tool need to be performed. In this regard, as shown in Table 1, the concentration of circulating histones in sepsis varies widely across different studies, ranging from nanograms per milliliter (ng·mL^−1^) to micrograms per milliliter (μg·mL^−1^). This variability highlights the challenge in establishing a standardized reference range for circulating histones as a diagnostic and prognostic marker in critically ill conditions, particularly in sepsis. Several studies have reported histone H3 levels in the range of ng·mL^−1^, such as Ito et al., where serum histone H3 concentrations above 9 ng·mL^−1^ were associated with DIC [89]. In contrast, other studies using different detection methods, such as ELISA or mass spectrometry, have found circulating histone levels exceeding 10 μg·mL^−1^ in septic shock patients, with values above 7.56 μg·mL^−1^ being predictive of mortality [85] (see Table 1). The heterogeneity in histone concentrations across studies may result from differences in detection methods, sample processing, patient populations, and disease severity. These discrepancies underscore the need for standardized quantification techniques and reference ranges, for both nucleosomes and circulating histones, to enhance the clinical usefulness of histones as reliable biomarkers for sepsis prognosis and prognostic.

When a cascade of events mediated by DAMPs occurs, adequate biomarkers and biomonitoring techniques are required to determine the optimal timing for appropriate therapeutic techniques used for the elimination of these DAMPs [111]. In this regard, MS offers a potential tool for quantifying circulating histones in sepsis and other critically ill conditions. Importantly, recent advances in MS‐based spatial epi‐proteomics allow the analysis of histone PTMs from low‐abundance clinical samples, enhancing biomarker identification in critical care settings [112].

Despite advancements in sepsis treatment, an optimal therapeutic approach remains elusive, largely due to the lack of well‐defined clinical endpoints and efficient biomarkers to monitor treatment success. Consequently, technologies aimed at assessing the efficacy of targeted therapies to neutralize histone toxicity are crucial for improving patient outcomes. Quantitative measurement of circulating histones in blood samples is emerging as a promising tool not only for the diagnosis and prognosis of sepsis, but also for evaluating the effectiveness of extracorporeal blood purification therapies, ensuring a more personalized and targeted approach to sepsis management [95]. Optimizing sample preparation and MS protocols to reduce the time required for measuring circulating histones may accelerate the application of this technique in clinical settings.

Personalized medicine for managing critically ill patients is evolving with the integration of extracorporeal blood purification devices that target key inflammatory mediators, including PAMPs and DAMPs. Devices engineered with matrices and materials capable of adsorbing circulating histones offer patient‐specific extracorporeal therapeutic options by selectively removing molecules that drive sepsis progression. In this context, circulating histone levels have emerged as potential biomarkers to guide personalized treatment strategies, helping to determine both the need for and selection of extracorporeal therapies. The integration of genetic and epigenetic signatures, molecular biomarkers, and real‐time inflammatory profiling, along with histone measurements, could refine precision‐based interventions, optimizing treatment efficacy and improving patient survival rates.

## Conflict of interest

JLG‐G is co‐founder and owns shares of EpiDisease S.L., a Spin‐Off of the Consortium Center for Biomedical Network Research of the ISCIII. EpiDisease S.L. has licensed the patent titled “Mass spectrometry‐based methods for the detection of circulating histones H3 and H2B in plasma from sepsis or septic shock (ss) patients” with reference number EP3535587B1, and extended to USA, China, Japan, and Hong Kong. The remaining authors declare no conflict of interest related to this work.

## Author contributions

JLGG, RF, AA, JCRR, and NCM conceived this review. RD, ASR, ECM, IB, DBL, AC, LCC, GGF, GG, VM, BQS, ARS, and ICC acquired the data, analyzed the data, prepared the table, and interpreted the data. JLGG, RF, AA, JCRR, and NCM wrote the paper and prepared the figures.
